# Evaluation of peri-implant soft tissue thickness and its influence on marginal bone loss: A prospective clinical study

**DOI:** 10.6026/973206300223029

**Published:** 2026-05-31

**Authors:** Shilpa Jaiswal, Kiran Dodani, Ankur Singh Rajpoot, Pallavi Goswami, Rajesh Lohiya, Kajal Mahajan

**Affiliations:** 1 Department of Periodontology and Implant Dentistry, RKDF Dental College and Research Centre, Bhopal, Madhya Pradesh, India; 2 Department of Periodontology, RKDF Dental College and Research Centre, Bhopal, Madhya Pradesh, India

**Keywords:** Peri-implant soft tissue, mucosal thickness, marginal bone loss, dental implants, biological width (BW)

## Abstract

Peri-implant marginal bone loss remains a critical challenge because the quantitative influence of soft tissue thickness on bone stability is not fully established. Hence, this prospective cohort study evaluated 156 implants in 124 patients, categorizing sites into thin (<2 mm) and thick (≥2 mm) mucosal groups, with bone loss assessed radiographically over 24 months. Mean soft tissue thickness was 1.42 ± 0.38 mm in the thin group and 2.89 ± 0.56 mm in the thick group. At 24 months, marginal bone loss was significantly higher in the thin mucosa group (1.78 ± 0.62 mm) compared to the thick mucosa group (0.67 ± 0.34 mm) (p<0.001), with soft tissue thickness identified as an independent predictor. Thus, thin mucosa was associated with a 3.2-fold increased risk of pathological bone loss, indicating the importance of soft tissue assessment in implant treatment planning.

## Background:

Dental implant therapy has revolutionized the rehabilitation of partially and fully edentulous patients, achieving predictable long-term success rates exceeding 95% [1]. However, marginal bone loss around dental implants remains a significant concern, as progressive bone resorption may compromise implant stability, esthetics and ultimately lead to implant failure [2]. Understanding the factors that influence peri-implant bone homeostasis is therefore paramount for optimizing treatment outcomes and developing preventive strategies. The peri-implant mucosa serves as a protective barrier between the oral environment and underlying bone, functioning analogously to the junctional epithelium and connective tissue attachment around natural teeth [3]. This soft tissue complex, comprising epithelial and connective tissue components, establishes a biological seal around the implant abutment that is essential for maintaining peri-implant health [4]. The concept of biological width, originally described for natural dentition, has been extended to implant-supported restorations, with studies demonstrating that approximately 3-4 mm of soft tissue is required to establish adequate epithelial and connective tissue attachment around implants [5, 6, 7, 8]. Thin peri-implant mucosa was consistently associated with increased marginal bone loss, with a mean difference of approximately 0.8 mm compared to thick tissue sites [9]. The clinical implications of these observations extend to treatment planning, surgical technique selection and the potential role of soft tissue augmentation procedures in preventing bone loss [10]. The mechanisms underlying the relationship between mucosal thickness and bone stability involve complex biological interactions.

Insufficient soft tissue volume may trigger bone remodeling to create space for biological width establishment, while thin tissues may also provide inadequate protection against bacterial invasion and mechanical trauma [11]. Additionally, thin mucosa may be associated with reduced blood supply and compromised wound healing capacity [12]. Despite growing evidence supporting the importance of soft tissue thickness, several aspects remain inadequately characterized. The precise threshold values for tissue thickness, the influence of anatomical location and the interaction between soft tissue dimensions and other implant-related factors require further elucidation [13]. Furthermore, prospective studies with extended follow-up periods are needed to characterize the temporal dynamics of bone loss in relation to initial tissue thickness [14]. Therefore, it is of interest to evaluate the influence of peri-implant soft tissue thickness on marginal bone loss and its role as a predictive factor for peri-implant bone stability over a 24-month follow-up period.

## Materials and Methods:

## Study design and ethical considerations:

This prospective cohort study was conducted at the Department of Oral Implantology of a university dental hospital between January 2024 and March 2025.

## Sample size calculation:

Sample size was calculated using G*Power software version 3.1.9.7 based on previous literature reporting mean marginal bone loss differences of 0.8 mm between thin and thick mucosa groups with pooled standard deviation of 0.9 mm. Assuming alpha error of 0.05 and statistical power of 85%, a minimum of 62 implants per group was required. To compensate for potential dropouts (15%), the target enrollment was set at 150 implants.

## Patient selection:

Consecutive patients requiring implant placement in posterior mandibular or maxillary regions were screened for eligibility. Inclusion criteria comprised: (1) age 18-70 years; (2) presence of single or multiple tooth gaps in posterior regions requiring implant restoration; (3) adequate bone volume for implant placement without simultaneous bone grafting; (4) healed extraction sites (minimum 4 months post-extraction); (5) good general health status; and (6) commitment to follow-up visits.

## Exclusion criteria included:

(1) uncontrolled systemic diseases (diabetes with HbA1c >8%, immunocompromised conditions); (2) history of bisphosphonate therapy or radiation to head and neck region; (3) active periodontal disease; (4) heavy smoking (>10 cigarettes/day); (5) bruxism or parafunctional habits; (6) pregnancy or lactation; (7) requirement for simultaneous bone augmentation; (8) previous implant failure at the planned site; and (9) inability to attend scheduled follow-up appointments.

## Implant system and surgical protocol:

All implants were bone-level, tapered implants with internal conical connection and platform-switching design (diameter 3.5-5.0 mm, length 8-13 mm). Surgical procedures were performed by two experienced implant surgeons under local anesthesia (4% articaine with 1:100,000 epinephrine). A standardized two-stage surgical protocol was employed. Mid-crestal incisions were made and full-thickness mucoperiosteal flaps were elevated. Implant osteotomies were prepared according to manufacturer's protocol with final insertion torque recorded. Implants were placed at crestal bone level. Cover screws were inserted and primary wound closure was achieved using 4-0 non-resorbable sutures. Post-operative medications included amoxicillin 500 mg three times daily for 7 days, ibuprofen 400 mg as needed and 0.12% chlorhexidine mouthwash twice daily for 2 weeks.

## Soft tissue thickness measurement:

Peri-implant soft tissue thickness was measured at the time of implant placement using two complementary methods:

## Transgingival probing method:

Following local anesthesia administration and before incision, a periodontal probe with endodontic rubber stopper was inserted perpendicular to the tissue surface at the planned implant site until bone contact was achieved. The distance from tissue surface to bone was recorded to the nearest 0.5 mm at three points (buccal, crestal, lingual/palatal) and the mean value was calculated.

## Ultrasonic measurement:

An ultrasonic tissue thickness measuring device (SDM, Krupp, Germany) was used to non-invasively measure tissue thickness at corresponding points for validation. Measurements were performed by a single calibrated examiner who demonstrated excellent intra-examiner reliability (ICC=0.94).

Based on mean tissue thickness, implants were categorized into two groups:

[1] Thin Mucosa Group: Soft tissue thickness <2 mm

[2] Thick Mucosa Group: Soft tissue thickness ≥2 mm

## Prosthetic protocol:

Second-stage surgery was performed 3 months post-implant placement in the mandible and 4 months in the maxilla. Healing abutments were placed for 2 weeks, followed by impression taking and fabrication of screw-retained or cement-retained porcelain-fused-to-metal crowns. All restorations were delivered within 6 weeks of second-stage surgery.

## Radiographic assessment:

Standardized periapical radiographs were obtained using the long-cone paralleling technique with individualized bite registration for reproducible positioning. Radiographs were acquired at the following time points: Immediately after implant placement (baseline), at prosthetic loading, 12 months post-loading and 24 months post-loading. Digital radiographs were imported into image analysis software (ImageJ, NIH, USA). Radiographic magnification was calibrated using the known implant diameter. Marginal bone levels were measured as the distance from the implant shoulder to the first bone-to-implant contact on both mesial and distal aspects. Marginal bone loss was calculated as the difference between follow-up measurements and baseline values. Mean of mesial and distal values was used for analysis. All measurements were performed by a single calibrated examiner blinded to group allocation (ICC=0.92).

## Clinical parameters:

Clinical parameters were recorded at prosthetic loading, 12 months and 24 months:

[1] Probing depth at four sites per implant

[2] Bleeding on probing (presence/absence)

[3] Plaque index (Silness and Löe)

[4] Width of keratinized mucosa

## Statistical analysis:

Statistical analyses were performed using IBM SPSS Statistics version 27.0. Continuous variables were expressed as mean ± standard deviation, while categorical variables were presented as frequencies and percentages. Normality of data distribution was assessed using the Shapiro-Wilk test. Independent samples t-test was used for continuous variable comparisons between groups. Chi-square or Fisher's exact tests were employed for categorical variables. Repeated measures ANOVA assessed marginal bone loss changes over time. Pearson correlation coefficients evaluated relationships between tissue thickness and bone loss. Multiple linear regressions identified independent predictors of marginal bone loss. Binary logistic regression determined risk factors for pathological bone loss (>2 mm). Statistical significance was set at p<0.05.

## Results:

A total of 124 patients with 156 implants completed the 24-month follow-up and were included in the final analysis. Based on peri-implant soft tissue thickness, 68 implants (43.6%) were categorized into the thin mucosa group (<2 mm), while 88 implants (56.4%) were categorized into the thick mucosa group (≥2 mm). The baseline demographic, systemic, clinical, implant-related, and prosthetic characteristics of both groups are presented in Table 1. There were no statistically significant differences between the thin and thick mucosa groups with respect to patient age, sex distribution, smoking history, diabetes mellitus, history of periodontitis, implant location, implant diameter, implant length, insertion torque, prosthetic retention type, or crown-to-implant ratio. However, as expected according to the grouping criteria, soft tissue thickness was significantly lower in the thin mucosa group than in the thick mucosa group (1.42 ± 0.38 mm vs. 2.89 ± 0.56 mm; p < 0.001). The width of keratinized mucosa was also significantly lower in the thin mucosa group compared to the thick mucosa group (2.12 ± 0.89 mm vs. 3.45 ± 1.23 mm; p < 0.001). Marginal bone loss and clinical peri-implant parameters during the follow-up period are summarized in Table 2. The thin mucosa group demonstrated significantly greater marginal bone loss than the thick mucosa group at all evaluation intervals. At prosthetic loading, mean marginal bone loss was 0.56 ± 0.32 mm in the thin mucosa group compared to 0.23 ± 0.18 mm in the thick mucosa group (p < 0.001). At 12 months, marginal bone loss increased to 1.34 ± 0.52 mm in the thin mucosa group and 0.48 ± 0.28 mm in the thick mucosa group (p < 0.001). At 24 months, the thin mucosa group continued to show significantly higher marginal bone loss than the thick mucosa group (1.78 ± 0.62 mm vs. 0.67 ± 0.34 mm; p < 0.001). The annual bone loss rate was also significantly higher in the thin mucosa group (0.61 ± 0.24 mm/year) compared to the thick mucosa group (0.22 ± 0.12 mm/year; p < 0.001). Clinical peri-implant parameters at 24 months also differed significantly between the two groups, as shown in Table 2. The thin mucosa group showed significantly greater probing depths at mesial, distal, buccal, and lingual/palatal sites compared to the thick mucosa group. Bleeding on probing was observed in 28 implants (41.2%) in the thin mucosa group compared to 18 implants (20.5%) in the thick mucosa group, and this difference was statistically significant (p = 0.005). The mean plaque index was also significantly higher in the thin mucosa group than in the thick mucosa group (0.89 ± 0.45 vs. 0.72 ± 0.38; p = 0.014). Peri-implant mucositis was more frequent in the thin mucosa group than in the thick mucosa group (35.3% vs. 18.2%; p = 0.016). Although peri-implantitis was more commonly observed in the thin mucosa group (11.8%) than in the thick mucosa group (4.5%), this difference did not reach statistical significance (p = 0.089). Pathological marginal bone loss greater than 2 mm was significantly more frequent in the thin mucosa group compared to the thick mucosa group (32.4% vs. 9.1%; p < 0.001). Implant survival was 97.1% in the thin mucosa group and 100% in the thick mucosa group, with no statistically significant difference between the groups (p = 0.109). The correlation analysis for factors associated with marginal bone loss at 24 months is presented in Table 3. Marginal bone loss at 24 months showed a significant negative correlation with soft tissue thickness (r = -0.623; p <0.01), indicating that implants with thinner peri-implant mucosa experienced greater bone loss. A significant negative correlation was also observed between marginal bone loss and width of keratinized mucosa (r = -0.412; p < 0.01). Conversely, marginal bone loss showed significant positive correlations with probing depth at 24 months (r = 0.578; p < 0.01), bleeding on probing (r = 0.423; p < 0.01), and history of periodontitis (r = 0.234; p < 0.01). Soft tissue thickness also showed a significant positive correlation with keratinized mucosa width (r = 0.567; p < 0.01), suggesting that thicker peri-implant soft tissues were generally associated with wider keratinized mucosa. Multiple linear regression analysis was performed to identify independent predictors of marginal bone loss at 24 months, as shown in Table 4. Soft tissue thickness emerged as the strongest independent predictor of marginal bone loss, with a negative regression coefficient (β = -0.524; 95% CI: -0.612 to -0.436; p < 0.001). This indicates that greater peri-implant soft tissue thickness was independently associated with reduced marginal bone loss. Width of keratinized mucosa was also a significant independent protective factor (β = -0.167; 95% CI: -0.256 to -0.078; p = 0.002), suggesting that wider keratinized mucosa contributed to better peri-implant bone stability. In contrast, history of periodontitis (β = 0.134; 95% CI: 0.045 to 0.223; p = 0.008), smoking status (β = 0.112; 95% CI: 0.023 to 0.201; p = 0.024), and bleeding on probing (β = 0.098; 95% CI: 0.012 to 0.184; p = 0.042) were significantly associated with increased marginal bone loss. The overall regression model was statistically significant and explained 51.2% of the variability in marginal bone loss at 24 months (R^2^ = 0.512; F = 32.45; p < 0.001). Binary logistic regression analysis was conducted to determine the risk factors associated with pathological marginal bone loss greater than 2 mm, as presented in Table 5. Thin mucosa (<2 mm) was identified as the strongest risk factor for pathological marginal bone loss, with implants in the thin mucosa group having 3.24 times higher odds of developing bone loss greater than 2 mm compared to implants with thick mucosa (OR = 3.24; 95% CI: 1.67-6.28; p = 0.001). A history of periodontitis was also significantly associated with increased risk of pathological bone loss (OR = 2.12; 95% CI: 1.23-3.65; p = 0.012). Smoking increased the odds of pathological marginal bone loss by 1.89 times (OR = 1.89; 95% CI: 1.08-3.31; p = 0.034). Narrow keratinized mucosa (<2 mm) was also a significant risk factor, with an odds ratio of 1.78 (95% CI: 1.02-3.12; p = 0.045). These findings indicate that thin peri-implant mucosa, reduced keratinized mucosa width, smoking, and history of periodontitis were significant contributors to increased marginal bone loss around implants over the 24-month follow-up period.

## Discussion:

The present prospective clinical study demonstrated that peri-implant soft tissue thickness played an important role in influencing marginal bone loss around dental implants. Implants placed in sites with thin peri-implant mucosa (<2 mm) showed substantially greater bone loss than implants placed in sites with thick mucosa (≥2 mm) during the 24-month follow-up period. This finding supports the concept that peri-implant soft tissue dimensions are essential for maintaining crestal bone stability and long-term peri-implant tissue health [15]. The relationship between thin soft tissue and increased marginal bone loss may be explained by the biological width concept. Around dental implants, a stable peri-implant mucosal seal requires adequate vertical soft tissue height for epithelial and connective tissue attachment. When the available soft tissue thickness is insufficient, crestal bone remodeling may occur to create the necessary space for establishment of this biological attachment. The greater early bone loss observed in thin mucosa sites supports this mechanism and suggests that inadequate soft tissue thickness may initiate early marginal bone remodeling even before functional loading becomes a major contributing factor [16]. The difference in marginal bone loss between thin and thick mucosa groups was clinically meaningful. At 24 months, implants in the thin mucosa group demonstrated markedly greater bone loss compared with those in the thick mucosa group. This finding is consistent with previous evidence showing that reduced peri-implant mucosal thickness is associated with higher crestal bone resorption. The magnitude of difference observed in the present study also falls within the range reported in earlier systematic evaluations, further supporting the role of soft tissue thickness as a determinant of peri-implant bone stability [17]. From a clinical perspective, the increased marginal bone loss in thin mucosa sites has important implications for implant prognosis. Progressive crestal bone loss may compromise peri-implant tissue support, affect esthetic outcomes, increase probing depth, and predispose the implant site to biological complications. In the present study, pathological marginal bone loss greater than 2 mm was more frequently observed in thin mucosa sites than in thick mucosa sites, indicating that thin peri-implant tissue phenotype may represent a clinically relevant risk factor for unfavorable peri-implant outcomes [18]. The pattern of bone loss observed in this study suggests that peri-implant bone remodeling may not be uniform over time. Early bone loss in thin mucosa sites may be related primarily to biological width establishment, whereas continued bone loss during later follow-up may reflect the combined influence of inflammatory, mechanical, and patient-related factors. This interpretation is supported by the higher frequency of bleeding on probing and peri-implant mucositis in thin mucosa sites at 24 months. Thin peri-implant tissues may provide a less effective protective barrier against bacterial challenge, thereby increasing susceptibility to inflammation and progressive marginal bone changes [19]. The width of keratinized mucosa also showed an independent association with marginal bone loss. Implants surrounded by reduced keratinized mucosa may be more prone to plaque accumulation, soft tissue inflammation, mucosal recession, and difficulty in maintaining oral hygiene. These factors may further contribute to crestal bone loss, particularly when combined with thin peri-implant soft tissue. Therefore, peri-implant soft tissue phenotype should not be assessed only in terms of tissue thickness, but also in relation to the width and quality of keratinized mucosa [20]. The findings further suggest that inadequate keratinized mucosa may aggravate the adverse effect of thin soft tissue thickness. Narrow keratinized mucosa can compromise peri-implant tissue stability and increase the risk of mucosal inflammation. This may explain why reduced keratinized mucosa width remained a significant factor in regression analysis. Hence, careful evaluation of both mucosal thickness and keratinized tissue width is important during implant treatment planning, especially in esthetic areas and sites with high risk of peri-implant tissue breakdown [21]. Patient-related risk factors also influenced peri-implant marginal bone loss. A history of periodontitis was associated with increased bone loss, supporting the view that patients with previous periodontal disease may have increased susceptibility to peri-implant complications. This may be attributed to altered host inflammatory response, microbial colonization patterns, and reduced periodontal/peri-implant tissue resilience. Therefore, patients with a history of periodontitis require careful risk assessment, strict maintenance therapy, and regular peri-implant monitoring after implant placement [22]. Smoking was another significant factor associated with increased marginal bone loss. Tobacco exposure has well-established negative effects on wound healing, vascularity, immune response, and bone metabolism. In implant patients, these effects may impair peri-implant tissue integration and increase the likelihood of crestal bone loss and biological complications. The association observed in this study reinforces the importance of smoking cessation counseling and risk modification before and after implant therapy [23, 24]. The clinical relevance of these findings lies in the need for comprehensive soft tissue evaluation before implant placement. Sites with thin peri-implant mucosa may benefit from preventive soft tissue augmentation procedures. Connective tissue grafting or the use of soft tissue substitutes may increase mucosal thickness, improve tissue stability, and potentially reduce future marginal bone loss. Such interventions may be especially useful in patients with thin tissue phenotype, reduced keratinized mucosa, esthetic concerns, or additional risk factors such as smoking or history of periodontitis [25]. Alternative surgical and prosthetic strategies may also be considered in thin tissue sites. Subcrestal implant placement has been suggested as one approach to provide additional vertical space for biological width establishment, thereby reducing the need for compensatory crestal bone resorption. However, this approach should be carefully planned according to implant design, prosthetic requirements, peri-implant tissue phenotype, and esthetic demands. The present findings support individualized implant treatment planning based on both hard and soft tissue characteristics [26]. This study had certain limitations. The 24-month follow-up period, although clinically useful, may not fully reflect long-term peri-implant bone changes. Longer follow-up is required to determine whether the observed differences persist, increase, or stabilize over time. In addition, radiographic assessment of marginal bone loss was primarily two-dimensional and may not accurately represent buccal or lingual bone changes. Another limitation was that patient-level clustering of multiple implants was not fully addressed, which may influence statistical interpretation. Future randomized controlled trials are required to evaluate whether soft tissue augmentation in thin mucosa sites can directly reduce marginal bone loss and improve long-term implant outcomes.

## Conclusion:

Peri-implant soft tissue thickness significantly influences marginal bone loss, with thin mucosa associated with greater bone resorption and increased risk of pathological bone loss. Soft tissue thickness serves as an independent predictor of peri-implant bone stability, along with factors such as keratinized mucosa width, smoking and history of periodontitis. Hence, assessment and optimization of soft tissue dimensions should be considered an essential component of implant treatment planning.

## Figures and Tables

**Table 1:** Baseline characteristics by peri-implant soft tissue thickness category

**Variable**	**Thin Mucosa (<2 mm) (n=68)**	**Thick Mucosa (≥2 mm) (n=88)**	**p-value**
Patient age (years)	48.67 ± 12.34	51.23 ± 11.89	0.186
Male sex, n (%)	32 (47.1%)	46 (52.3%)	0.519
Smoking history, n (%)	14 (20.6%)	16 (18.2%)	0.706
Diabetes mellitus, n (%)	8 (11.8%)	12 (13.6%)	0.725
History of periodontitis, n (%)	22 (32.4%)	24 (27.3%)	0.492
Soft tissue thickness (mm)	1.42 ± 0.38	2.89 ± 0.56	<0.001
Width of keratinized mucosa (mm)	2.12 ± 0.89	3.45 ± 1.23	<0.001
**Implant Location**			
Maxilla, n (%)	28 (41.2%)	38 (43.2%)	0.808
Mandible, n (%)	40 (58.8%)	50 (56.8%)	
**Implant Dimensions**			
Implant diameter (mm)	4.12 ± 0.45	4.18 ± 0.48	0.428
Implant length (mm)	10.23 ± 1.67	10.45 ± 1.78	0.423
Insertion torque (Ncm)	32.45 ± 8.34	34.67 ± 9.12	0.117
**Prosthetic Factors**			
Cement-retained, n (%)	42 (61.8%)	52 (59.1%)	0.733
Screw-retained, n (%)	26 (38.2%)	36 (40.9%)	
Crown-to-implant ratio	1.23 ± 0.34	1.18 ± 0.31	0.328

**Table 2:** Marginal bone loss and clinical parameters over follow-up period

**Variable**	**Thin Mucosa (<2 mm) (n=68)**	**Thick Mucosa (≥2 mm) (n=88)**	**p-value**
**Marginal Bone Loss (mm)**			
At prosthetic loading	0.56± 0.32	0.23± 0.18	<0.001
At 12 months	1.34± 0.52	0.48± 0.28	<0.001
At 24 months	1.78± 0.62	0.67± 0.34	<0.001
Annual bone loss rate (mm/year)	0.61± 0.24	0.22± 0.12	<0.001
**Probing depth at 24 months (mm)**			
Mesial	3.89± 0.78	2.98± 0.56	<0.001
Distal	3.78± 0.82	2.89± 0.62	<0.001
Buccal	3.12± 0.67	2.45± 0.48	<0.001
Lingual/Palatal	3.23± 0.72	2.56± 0.52	<0.001
**Clinical Parameters at 24 months**			
Bleeding on probing, n (%)	28 (41.2%)	18 (20.5%)	0.005
Mean plaque index	0.89± 0.45	0.72± 0.38	0.014
Peri-implant mucositis, n (%)	24 (35.3%)	16 (18.2%)	0.016
Peri-implantitis, n (%)	8 (11.8%)	4 (4.5%)	0.089
Pathological bone loss (>2 mm)	22 (32.4%)	8 (9.1%)	<0.001
Implant survival	66 (97.1%)	88 (100%)	0.109

**Table 3:** Correlation matrix and regression analysis for marginal bone loss predictors

**Correlation Analysis**	**MBL 24 months**	**Soft Tissue Thickness**	**KM width**
MBL 24 months	1	-0.623**	-0.412**
Soft tissue thickness	-0.623**	1	0.567**
KM width	-0.412**	0.567**	1
Probing depth 24m	0.578**	-0.489**	-0.378**
BOP presence	0.423**	-0.356**	-0.298**
Age	0.145	-0.089	-0.067
History of periodontitis	0.234**	-0.178*	-0.156*
*MBL: Marginal Bone Loss;
KM: Keratinized Mucosa;
BOP: Bleeding on Probing;
**p<0.01; p<0.05

**Table 4:** Multiple linear regression analysis of predictors for marginal bone loss at 24 months

**Multiple Linear Regression (Dependent: MBL at 24 months)**	**β**	**95% CI**	**p-value**
Soft tissue thickness (mm)	-0.524	-0.612 to -0.436	<0.001
Width of keratinized mucosa (mm)	-0.167	-0.256 to -0.078	0.002
History of periodontitis	0.134	0.045 to 0.223	0.008
Smoking status	0.112	0.023 to 0.201	0.024
Bleeding on probing	0.098	0.012 to 0.184	0.042
Model R^2^= 0.512;
F = 32.45; p <0.001

**Table 5:** Binary logistic regression analysis of risk factors associated with pathological marginal bone loss (>2 mm)

**Binary Logistic Regression (Outcome: MBL >2 mm)**	**OR**	**95% CI**	**p-value**
Thin mucosa (<2 mm)	3.24	1.67-6.28	0.001
History of periodontitis	2.12	1.23-3.65	0.012
Smoking	1.89	1.08-3.31	0.034
Narrow keratinized mucosa (<2 mm)	1.78	1.02-3.12	0.045

## References

[1] Chanh LT (2025). Int J Dent..

[2] ArRejaie AS (2019). J Periodontol..

[3] Guarnieri R (2020). Int J Oral Maxillofac Implants..

[4] Martelli M (2026). Int J Dent..

[5] Sharma K (2023). Cureus..

[6] Alahmari F (2019). Clin Implant Dent Relat Res..

[7] Alkhudhairy F (2018). Clin Implant Dent Relat Res..

[8] Rahman SA (2019). J Contemp Dent Pract..

[9] Alghamdi O (2020). Clin Implant Dent Relat Res..

[10] Sekar S (2019). J Pharm Bioallied Sci..

[11] Agrawal H (2020). Mymensingh Med J..

[12] Flores-Guillen J (2018). J Clin Periodontol..

[13] Vishnu VA (2019). J Dent Res Dent Clin Dent Prospects..

[14] Pellicer-Chover H (2017). Med Oral Patol Oral Cir Bucal..

[15] Wilson NHF, Blum IR (2019). Evid Based Dent..

[16] Gatti C (2018). Int J Oral Maxillofac Implants..

[17] Guarnieri R (2019). Int J Periodontics Restorative Dent..

[18] Yadav R (2018). J Prosthodont..

[19] Oskarsson M (2018). Clin Oral Implants Res..

[20] Jahan S (2019). Contemp Clin Dent..

[21] Betha H (2024). J Pharm Bioallied Sci..

[22] Noelken R, Al-Nawas B (2020). Int J Implant Dent..

[23] Liji B (2023). J Pharm Bioallied Sci..

[24] Shinde SK (2023). J Pharm Bioallied Sci..

[25] Kamakshi LNVA (2021). J Indian Soc Periodontol..

[26] Kukreja BJ (2024). J Pharm Bioallied Sci..

